# Serum Chemerin Is Decreased by Roux-en-Y Gastric Bypass and Low Calorie-Formula Diet in Obese Individuals

**DOI:** 10.3390/biomedicines12010033

**Published:** 2023-12-22

**Authors:** Andreas Schmid, Martin Roderfeld, Thomas Karrasch, Elke Roeb, Andreas Schäffler

**Affiliations:** 1 Department of Internal Medicine III, Justus Liebig University, 35392 Giessen, Germany; 2 Department of Gastroenterology, Justus Liebig University, Klinikstr. 33, 35392 Giessen, Germany

**Keywords:** chemerin, obesity, weight loss, Roux-en-Y gastric bypass, low calorie-formula diet

## Abstract

The pleiotropic chemokine chemerin is involved in multiple processes in metabolism and inflammation. The present study aimed to elucidate its regulation in morbid obesity and during therapy-induced rapid weight loss. A total of 128 severely obese patients were enrolled, and their basal anthropometric and clinical parameters were assessed. In total, 64 individuals attended a conservative 12-month weight loss program that included a low calorie-formula diet (LCD), and 64 patients underwent bariatric surgery (Roux-en-Y gastric bypass, RYGB). Blood serum was obtained at study baseline and at follow-up visits after 3, 6, and 12 months. Systemic chemerin concentrations, as well as metabolic and immunological parameters, were quantified. During the 12-month period studied, serum chemerin levels decreased significantly with weight loss after bariatric surgery, as well as with conservative low calorie therapy; however, the effects of RYGB were generally stronger. No substantial associations of systemic chemerin concentrations with therapy-induced improvement of type 2 diabetes and with indicators of liver function and fibrosis were observed. We conclude that systemic chemerin levels decrease in obese individuals during weight loss, regardless of the therapeutic strategy. A potential involvement in weight loss-associated improvement of metabolic disorders and liver fibrosis remains to be further investigated.

## 1. Introduction

Metabolic syndrome—comprising obesity, together with a number of co-morbidities and metabolic disorders, such as type 2 diabetes mellitus (T2D), dyslipidemia, and hypertension—represents a major problem for public health worldwide, with growing prevalence [1]. On the cross-roads of metabolic dysregulation and immunity, metaflammation acts as a substantial mechanism of the metabolic syndrome [2]. Besides these severe health issues originating from obesity per se, metabolic-associated steatotic liver disease (MASLD)—with phenotypes including steatohepatitis and liver fibrosis—are of increasing relevance [3,4], and their prevalence is clearly associated with obesity [5].

Among the established therapies for the induction of sustained weight loss and curation of concomitant metabolic disorders, dietary approaches represent a widely applied conservative option [6]. As an alternative to dietary and lifestyle intervention, bariatric surgery strategies comprise potent therapeutical tools enabling weight loss, fat mass reduction, and beneficial metabolic outcome in severely obese individuals [7]. Importantly, recent years have witnessed increasing efforts in order to optimize the individual allocation of patients to anti-obesity therapy options [8]. Against this background, we previously presented data from a large comparative study comprising obese patients either undergoing bariatric (Roux-en-Y (RYGB) or gastric sleeve) surgery or attending a low calorie-formula diet (LCD) program [9,10]. Circulating quantities of progranulin and C1q/TNF-related protein 3 (CTRP3) have previously been investigated in this study cohort. While a positive correlation between serum quantities of both adipokines was detected, rather contrary kinetics were observed during weight loss, with a significant increase in progranulin and a decrease in CTRP3 concentrations within 12 months [9,10].

A number of secretory proteins derived from adipose tissue—generally referred to as adipokines—are affected by and contribute to the mechanisms underlying obesity-related inflammation [11]. Due to its involvement in multiple entities of metabolic dysregulation [12], the adipokine chemerin has been suggested as a biomarker for the metabolic syndrome [13]. It is predominantly expressed in adipocytes [14] and hepatocytes [15], alongside lower expression levels in further organs, as a product of the *RARRES2* gene. The initially synthesized and secreted pro-chemerin (143 amino acids) is further processed, undergoing subsequent cleavage events that are catalyzed by extracellular proteases and regulate the bioactivity of mature chemerin [16]. Activated chemerin isoforms as chemoattractant factors have an essential part in the onset and regulation of tissue inflammation via modulation of immune cell recruitment and chemotaxis [12]. Of note, chemerin exhibits both pro- and anti-inflammatory properties. For instance, the hepatic overexpression of chemerin was shown to be protective against inflammation in non-alcoholic steatohepatitis [17]. Exceeding its role as an immune-modulatory adipokine, chemerin is significantly involved in various metabolic processes [12]. Of note, elevated systemic chemerin concentrations alongside a negative correlation with insulin sensitivity in obesity have recently been reported, suggesting that the relation of serum chemerin to glucose metabolism might depend on overweight [18].

Overall, the results from the present literature on chemerin’s precise role within the metabolic syndrome remains somewhat inconsistent. Our present approach aimed to investigate the relation of chemerin to metabolic disorders associated with morbid obesity, T2D, arterial hypertension, dyslipidemia, and liver fibrosis. In particular, the regulation of systemic chemerin in morbidly obese individuals under the conditions of weight and fat loss represents a highly relevant yet so far unknown issue remaining to be elucidated by current clinical studies. Our main goal therefore was to investigate chemerin kinetics during significant weight loss and its interrelation with a beneficial metabolic outcome in the context of the aforementioned disorders. Of note, the present study included a comparative investigation of chemerin kinetics during and after conservative and bariatric intervention, thus enabling a more distinguished interpretation of observed effects and correlations, as was performed in recent studies [9,10].

In order to address the aforementioned issues, the study encompassed a total of 128 severely obese patients undergoing either RYGB surgery or LCD. Our investigation focused on the following aspects:

- Regulation of circulating chemerin in severe obesity and its correlation with anthropometric, inflammatory, and metabolic parameters;
- Comparative investigation of weight loss-associated effects on chemerin driven by either bariatric surgery (RYGB) or conservative therapy (LCD);
- Elucidation of chemerin as a biomarker of metabolic disorders and as a predictor of an ameliorated metabolic-inflammatory state as a consequence of weight loss.

## 2. Materials and Methods

### 2.1. ROBS (Research in Obesity and Bariatric Surgery) Study Cohort

Blood serum was collected from severely obese individuals participating in the *ROBS* (Research in Obesity and Bariatric Surgery) study, which was previously introduced in detail [9]. As a longitudinal and observational study at the University Hospital Giessen, Germany, *ROBS* includes patients routinely undergoing either bariatric surgery (gastric sleeve or Roux-en-Y gastric bypass) or a low calorie-formula diet (LCD) as a dietary therapy approach for weight loss. Patients participating in the study must not meet the exclusion criteria of pregnancy, underlying endocrine diseases, untreated bulimia nervosa and binge eating behavior, illicit drug abuse, neoplasm, severe psychiatric disorders, psychosis, and psychopathologic instability.

The 12-month dietary regimen applied for the LCD sub-cohort included an initial period of 12 weeks of restricting nutrition to a balanced formula diet (~900 kcal/day), followed by successive replacement of formula nutrition by conventional meals within 8 weeks, and a final stabilizing period without any formula nutrition.

For the present investigations, serum samples and previously assessed anthropometric and biochemical data from study time points V0 (baseline), V3, V6, and V12 (routine medical 3-, 6-, and 12-month follow-up visits) of *ROBS* subjects were analyzed. These follow-up examination time points were chosen due to the observed considerable decrease in body weight and fat content within both study cohorts during the respective time intervals [9,19] and, additionally, in order to identify the potential effects of the changing nutritional conditions during LCD.

The present study investigated a subset of the previously introduced clinical cohort [9] that was approved by the local ethical committee at the University of Giessen, Germany (file code: *AZ 101/14*). All patients gave their informed consent, and data anonymization and a privacy policy were applied accurately.

In the present subset, 79 patients of RYGB surgery and 81 individuals undergoing LCD were studied. Alongside general anthropometric and physiological data, scores of liver fibrosis (NFS, FIB-4) were assessed according to the calculation formula [20,21,22]. More detailed information concerning this *ROBS* subset can be retrieved from a recent publication [19].

### 2.2. Data Collection

Samples and the general data of patients were obtained at study baseline and at routine follow-up visits 3, 6, and 12 months after bariatric surgery or the beginning of dietary therapy, respectively. General anthropometric, clinical, and psychological data, as well as medication, smoking habits, nutritional status, and routine laboratory data, were assessed and were reported previously [19]. Patient subgroups with advanced (NFS > 0.675) and without liver fibrosis (NFS < −1.455) were determined, as well as those with and without T2D.

### 2.3. Quantification of Circulating Chemerin Concentrations

Venous blood was drawn from all study subjects at the time points V0, V3, V6, and V12, and serum was obtained via centrifugation (4000 rpm, 15 min, and 4 °C). Serum chemerin quantities were measured by applying enzyme-linked immunosorbent assay (ELISA) techniques (DuoSet ELISA development kits, R&D systems, Wiesbaden, Germany) in technical duplicates. Quantification was repeated whenever intra-duplicate variation exceeded 20% in a measurement.

### 2.4. Statistical Analysis

Data analysis was performed applying the statistical software package *SPSS 27.0* (Armonk, IBM Statistics, NY, USA). Data were tested for normal distribution by applying the Wilk–Shapiro test. For the analysis of numerical parameters, the non-parametric *Mann–Whitney* U test (for 2 unrelated groups) or *Wilcoxon* test (for 2 related groups) was applied. Dynamic changes were tested with a general linear model for repeated measures. Distribution of categorial variables was analyzed by applying the McNemar test (for related samples). The non-parametric *Spearman’s rho* test was applied for the correlation analysis. A receiver operation characteristic (ROC) curve analysis was applied for the numerical parameters as potential determinants of classified variables. In general, a *p*-value below 0.05 (two-tailed) was considered statistically significant. Data are graphically presented either with dots representing mean values and whiskers giving the standard error of the mean (SEM); or by box plots displaying median, upper/lower quartiles, interquartile range, and outliers which are indicated as dots and asterisks.

## 3. Results

### 3.1. Study Cohort Characteristics

Standard anthropometric and physiological data of the ROBS study cohort as a whole were published recently [9,19]. In the present study, the LCD sub-cohort comprised 25 men and 39 women (mean age 42.6 years), and 11 men and 53 women were included in the RYGB sub-cohort (mean age 40.2 years). A total number of 29 patients suffered from manifested T2D (11 in LCD and 18 in RYGB subgroup). The baseline BMI ranged from 33.7 to 61.2 kg/m^2^, with a significantly higher mean value in the bariatric subgroup (51.5 kg/m^2^; *n* = 64) when compared to LCD patients (43.8 kg/m^2^; *n* = 64) (*p* < 0.001). Both therapy strategies induced a significant weight loss within 12 months although RYGB proved to be more effective regarding the extent of body weight and BMI reduction than LCD. In addition to weight loss, a general amelioration of the metabolic state was observed predominantly in the bariatric sub-cohort at the 12 month follow-up, as was displayed by the reduced body fat proportion (RYGB: *p* < 0.001; LCD: *p* < 0.001) and waist–hip ratio (RYGB: *p* < 0.001; LCD: *p* < 0.001), as well as by improved type 2 diabetes mellitus (RYGB: *p* < 0.001), hypertension (RYGB: *p* < 0.001; LCD: *p* = 0.012), and hyperlipidemia ((RYGB: *p* < 0.001; LCD: *p* = 0.013).

### 3.2. Systemic Chemerin Levels at Study Baseline

Serum chemerin was quantified in all study subjects. Baseline concentrations ranged from 75.9 to 283.5 ng/mL (mean: 152.7 ± 54.8 ng/mL) in LCD participants and from 71.2 to 357.1 ng/mL (mean: 152.2 ± 54.2 ng/mL) in patients undergoing RYGB surgery. In both sub-cohorts, the baseline chemerin levels were not normally distributed and exhibited positive skewness. Regarding the total study cohort, a slight difference in chemerin levels between male and female patients (140.7 vs 157.1 ng/mL) was observed as a non-significant trend.

**Figure 1:**
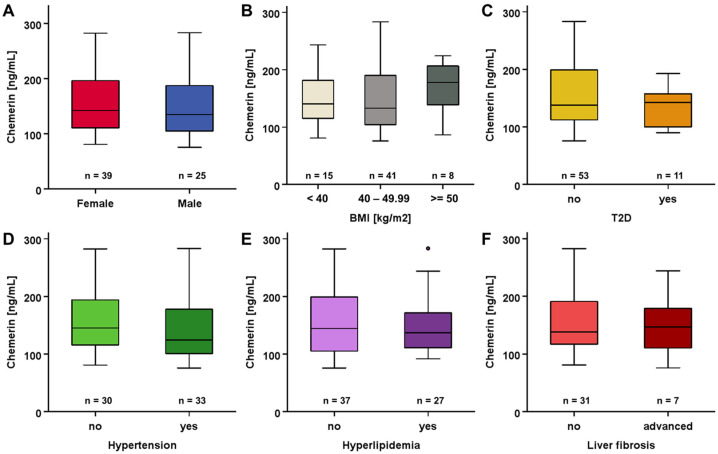
(**A**–**F**) Circulating chemerin concentrations in patients undergoing LCD were not affected by differences in sex, BMI, hypertension, T2D, hyperlipidemia, and liver fibrosis at study baseline.

Focusing on the cohort of patients receiving LCD therapy, we observed no sexual dimorphism in regard to baseline chemerin levels (Figure 1A). There was also no significant divergence with regard to BMI (Figure 1B). The further subgroup analysis involving patients’ medical history did not indicate any substantial impact of T2D, hypertension, hyperlipidemia, and liver fibrosis on systemic chemerin concentrations (Figure 1C–F).

**Figure 2:**
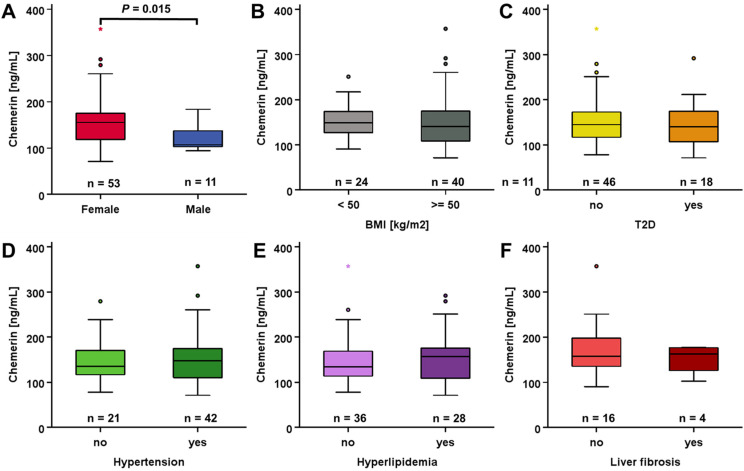
Pre-surgery systemic chemerin concentrations were elevated in female RYGB patients (**A**), whereas BMI, hypertension, T2D, hyperlipidemia, and liver fibrosis had no effect (**B**–**F**).

Among patients being allocated to RYGB surgery, females exhibited higher baseline systemic chemerin levels (158.7 ng/mL) compared to males (121.0 ng/mL) (Figure 2A). BMI, T2D, hypertension, hyperlipidemia, and liver fibrosis apparently did not significantly affect chemerin quantities (Figure 2B–F).

**Figure 3:**
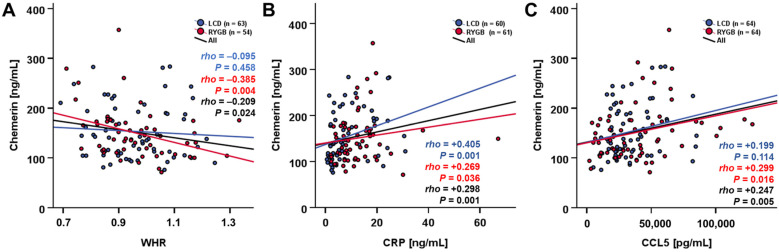
At study baseline, serum chemerin levels were correlated with WHR, CRP, and CCL5 in RYGB patients (**A**–**C**).

Regarding the whole study cohort (n = 128), serum chemerin quantities were negatively correlated with the waist–hip ratio (*rho* = −0.209, *p* = 0.024) and positively correlated with CRP (*rho* = 0.298, *p* = 0.001) and CCL5 levels (*rho* = 0.247, *p* = 0.005) (Figure 3).

### 3.3. Changes in Chemerin Levels during Weight Loss

Upon bariatric surgery or during caloric restriction, respectively, patients experienced significant and prolonged weight loss within 12 months, as was reported recently [19].

**Figure 4:**
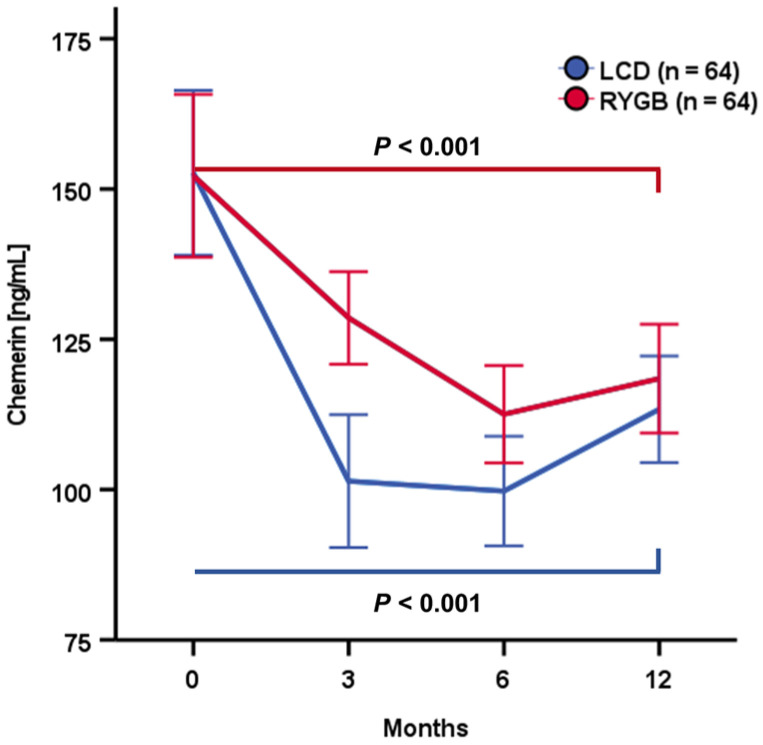
Serum chemerin concentrations substantially decreased during the initial phase of weight loss and remained at lower levels after 12 months (V12).

Figure 4 displays the dynamics of systemic chemerin concentrations during this period of weight loss for both study cohorts. Both RYGB surgery and LCD induced a considerable decline in chemerin levels within the initial 3–6 months upon intervention/therapy start, with a more rapid and pronounced decrease among LCD patients (~35% reduction within 3 months). Albeit a subsequent slight recovery, chemerin concentrations remained at a significantly lower level after 12 months when compared to baseline quantities. In both sub-cohorts, no significant divergences of chemerin kinetics in non-diabetic and T2D patients were detected.

### 3.4. ROC and Correlation Analysis of Chemerin Levels and Improvement of Metabolic Parameters

**Figure 5:**
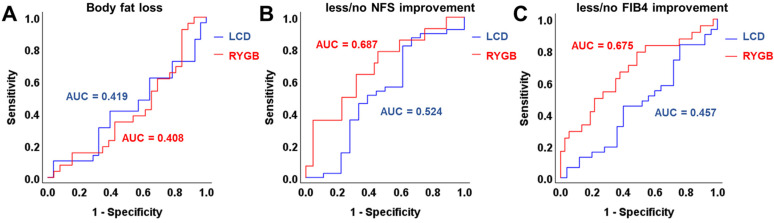
ROC analysis of the predictive potency of baseline chemerin levels for beneficial changes in body fat percentage (**A**), NFS (**B**), and FIB4 (**C**) during weight loss in LCD and RYGB patients.

A receiver operating characteristic (ROC) curve analysis was applied in order to identify a potential predictive quality of systemic chemerin concentrations for changes in metabolic dysregulation during weight loss (Figure 5). The results indicate that baseline chemerin quantities were not predictive for weight loss-associated dynamics of body fat percentage (Figure 5A) and liver fibrosis—as was displayed by the NAFLD fibrosis score and FIB4 (Figure 5B,C)—in LCD patients within 12 months. Among individuals undergoing RYGB surgery, a moderate association of basal chemerin levels with less/absent NFS and FIB4 improvement during weight loss was detected (Figure 5B,C).

**Figure 6:**
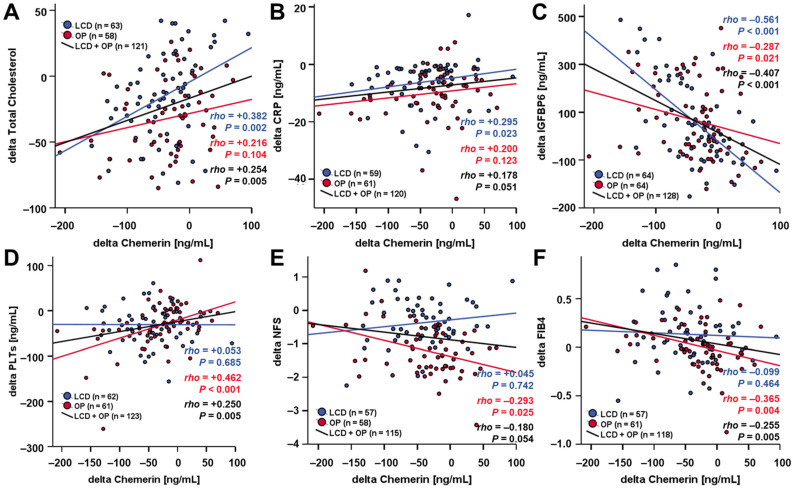
Changes in chemerin levels during weight loss are correlated with dynamics of total cholesterol (**A**), CRP (**B**), IGFBP6 (**C**), PLT (**D**), NFS (**E**), and FIB4 (**F**) over 12 months. CRP, C-reactive protein; FIB4, fibrosis-4 index; IGFBP6, insulin-like growth factor binding protein 6; NFS, NAFLD fibrosis score; PLTs, platelets.

The comparative correlation analysis revealed significant positive correlations of changes in systemic chemerin concentrations with total cholesterol and CRP dynamics during 12 months of weight loss in LCD but not in RYGB patients (Figure 6A,B). Furthermore, a considerable negative correlation of chemerin and IGFBP6 levels was observed that was more pronounced within the LCD subgroup (Figure 6C). On the other hand, a positive correlation of chemerin dynamics with ΔPLTs (changes in systemic platelets concentrations) and negative correlations with ΔNFS and ΔFIB4 were exclusively detected among RYGB patients (Figure 6D–F).

**Figure 7:**
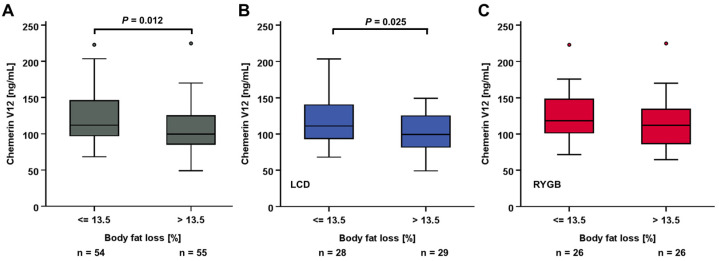
Serum chemerin concentrations after 12 months were decreased in patients experiencing a more pronounced loss of proportional body fat (**A**). This divergence was specifically observed in LCD patients (**B**), while it was absent in weight loss achieved via RYGB (**C**).

A further subgroup analysis comparing patients with differentially pronounced losses in body fat percentage (=<13.5% or >13.5%) revealed lower chemerin serum concentrations after 12 months among those individuals with higher fat loss (Figure 7A). Of note, this significant difference was exclusively observed among patients receiving LCD therapy but not among those having undergone RYGB surgery (Figure 7B,C).

## 4. Discussion

Previous studies reported elevated systemic chemerin levels in obesity, as well as a decline occurring during weight loss [18]. We provide data from a large and well-characterized obesity cohort, comprising patients undergoing either conservative or surgical treatment (n = 64 each) for body weight and fat reduction and therefore enabling a direct comparison of the effects induced by these competitive therapeutical strategies. Both the LCD intervention and RYGB surgery succeeded in significant weight loss and body fat reduction, as well as in metabolic improvement within one year, as reported recently [9,19]. In the present study, we observed an initial decline and sustained reduction in circulating chemerin concentrations accompanying weight loss throughout 12 months, with only slight differences in the outcomes of the RYGB and LCD sub-cohorts. Within the whole study cohort of 128 obese individuals, we assessed no significant sexual dimorphism in systemic chemerin concentrations, regarding levels at study baseline, as well as therapy-induced kinetics.

Furthermore, we did not observe any significant association of basal circulating chemerin levels with the extent of obesity and with common metabolic comorbidities such as T2D, hypertension, hyperlipidemia, and markers of hepatic fibrosis. Our data thus did not confirm the results of previous studies suggesting an association of chemerin with impaired glycemic control, as was summarized by a recent review [23]. However, the absence of significant correlations might be due to the low proportion of diabetic patients in the present study cohort (29 among 128 subjects). Among the anthropometric parameters assessed, the waist–hip ratio was negatively correlated with chemerin quantities. Of note, this correlation was exclusively found among the patients who were allocated to RYGB surgery. This sub-cohort exhibited a significantly elevated mean BMI (51.5 kg/m^2^) when compared to the group of LCD participants (43.8 kg/m^2^) at study baseline, while not differing according to the mean waist–hip ratio. The observed correlation therefore argues for a putative negative impact of high quantities of visceral adipose tissue—indicated by an elevated waist–hip ratio—on systemic chemerin concentrations predominantly occurring at an advanced extent of obesity. Of particular interest in this context, lowered chemerin expression levels in the visceral adipose tissue of obese individuals correlate with hepatic steatosis [24], suggesting the significant role of chemerin secreted from this adipose compartment during metabolic regulation in severe adiposity.

Of particular interest, serum chemerin concentrations were positively correlated with CRP and CCL5 levels, markers of systemic inflammation, within the whole study cohort. This finding is in good accordance with the significant involvement of chemerin in obesity-related inflammatory processes, acting as a chemoattractant protein [25,26]. The stronger correlation with CRP among the LCD sub-cohort—characterized by lower mean BMI—suggests that this association with systemic inflammation might be more pronounced in less severe obesity.

Two previous studies investigating the long-term impact of different bariatric procedures on circulating adipokine concentrations reported a significant long-term reduction in chemerin levels within 12 months after surgery [27,28]. To the best of our knowledge, our present approach is the first to directly compare the effects of conservative and bariatric interventions on systemic chemerin regulation in a large cohort of morbidly obese individuals. During the 12-month period of substantial body weight and fat loss—either following RYGB surgery or under a low-calorie diet—we observed a significant and largely sustained decrease in circulating chemerin quantities. Of note, LCD induced a more pronounced and rapid decline in serum chemerin, resulting in a reduction in circulating levels by ~35% within three months. The chemerin decrease during RYGB-induced weight loss occurred less rapidly, with the detected minimum of ~70% of baseline quantities being reached after 6 months. It therefore appears reasonable to conclude that a dietary shift to caloric restriction might affect serum chemerin in a faster and stronger way than bariatric surgery with subsequent limitations on food intake and nutrient absorption. Nevertheless, both weight loss strategies resulted in a significant and prolonged chemerin reduction, with some indication of slight recovery after 12 months. Since the latter finding implies a rather transient nature of the observed decline, it should motivate future longitudinal approaches in order to investigate circulating chemerin kinetics during long-term periods of conservative and surgical obesity therapy in relation to sustained weight loss and improvement in metabolic disorders.

Circulating chemerin has been characterized as a risk factor for NAFLD/MASLD [29] and has been suggested as a potential non-invasive biomarker [30]. We recently reported the correlation of body fat loss and improvement of liver fibrosis risk during obesity therapy [19]. Regarding the potential of systemic chemerin as a pre-interventional predictor of therapy-induced beneficial effects on metabolism, we did not observe any predictive potential of baseline systemic levels concerning a reduction in body fat percentage in the present study cohort. In the RYGB sub-cohort, a moderate predictive power of basal chemerin for less improvement of hepatic fibrosis—indicated by NFS and FIB4 index—was detected which was absent among LCD patients. Thus, the weight loss-associated amelioration of liver health and integrity upon bariatric surgery might rather be favored by lower baseline systemic chemerin concentrations. Such an association remains to be carefully verified by applying adequate study cohorts with a more predominant focus on these hepatic effects of obesity therapy.

Among patients attending the LCD program, we detected positive correlations of serum chemerin kinetics with changes in the total cholesterol and CRP level within the 12-month study period. This finding suggests an association of decreasing chemerin quantities with a reduction in circulating total cholesterol and CRP—hence, with an amelioration of the systemic metabolic and inflammatory state—that might be more pronounced during weight loss induced by caloric restriction than by bariatric surgery. A substantial negative correlation between the kinetics of chemerin and IGFBP6 levels was predominantly detected among LCD patients. Since elevated IGFBP6 concentrations are considered to be associated with hepatic steatosis [31], this observation might imply a rather inverse correlation between weight loss-associated chemerin decline and improvement in hepatic metabolism, especially under caloric restriction—whereas chemerin concentrations at study baseline were not predictive for LCD-induced subsequent improvement in hepatic fibrosis, as was mentioned above.

**Figure 8:**
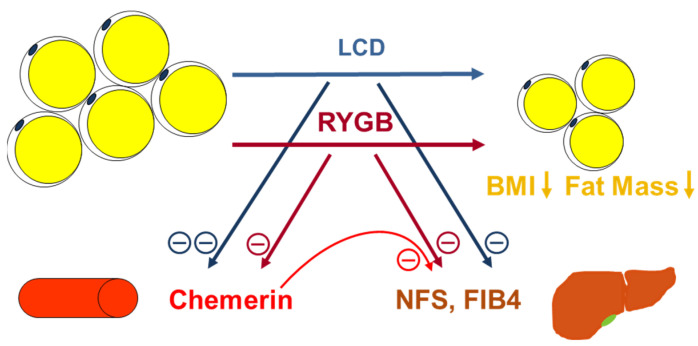
Circulating chemerin concentrations significantly decline during therapy-induced loss of body weight, BMI, and fat mass. The downregulation appears to be more pronounced under LCD than after RYGB surgery. Furthermore, reductions in body weight and fat mass are partially accompanied by a decrease in NFS [32] and FIB4 values, indicating reduced liver fibrosis risk. These changes in NFS and FIB4 during RYGB-induced weight loss are negatively correlated with chemerin kinetics, suggesting the hypothesis of chemerin being involved in the process of hepatic amelioration during post-surgical weight loss.

Patients undergoing RYGB surgery exhibited a positive correlation between post-surgical changes in chemerin and platelet concentrations, whereas negative correlations were observed with changes in NFS and FIB4. The latter finding suggests the hypothesis that, during RYGB-induced weight loss, chemerin might be involved in the amelioration of obesity-related hepatic fibrosis occurring during RYGB-induced weight loss, presumably in a protective role, as is illustrated in Figure 8.

When comparing systemic chemerin concentrations at the end of the 12-month study period in patients with a lower and higher pronounced reduction in body fat percentage (below and above the median value of reduction by 13.5%), we found higher body fat loss to be associated with lower chemerin levels. This effect was specific to patients attending the LCD program who experienced a more pronounced decline in chemerin levels during weight loss, as mentioned above. Besides potentially displaying reduced fat mass as a significant sign of chemerin expression, this finding might also indicate a lower level of remaining adipose inflammation in patients having experienced a stronger weight loss. Thus, our data might also underline the role of chemerin as an immune–metabolic factor linking mechanisms of obesity and inflammation [25].

The present study provides data on a large and well-characterized study cohort in a longitudinal setting, demonstrating the significant down-regulating impact of considerable weight loss on systemic chemerin quantities in severely obese individuals. Of particular interest is the comparative investigation of chemerin kinetics under dietary intervention and upon bariatric surgery, respectively, in order to distinguish effects associated with general weight loss from such induced by therapy-specific confounders.

The implications to be drawn from the study are limited by the focus on systemic chemerin quantities without elucidating the effects on chemerin expression in organs such as particular adipose tissue and the liver. Furthermore, the clinical relevance of the hypothesized role of chemerin as a biomarker for the weight loss-induced amelioration of liver integrity remains to be validated by studies involving cohorts with a higher proportion of patients with fibrosis or hepatic malfunction. The present design did not include control groups of non-obese individuals or of morbidly obese individuals not undergoing weight loss therapy in order to enable a comparative analysis of systemic chemerin. Finally, the molecular mechanisms underlying the observed chemerin kinetics during weight loss remain to be elucidated and should motivate further research focusing on molecular and cellular levels.

## 5. Conclusions

The present data analyses demonstrate a significant and prolonged decline in serum chemerin concentrations in severely obese individuals during therapy-induced weight loss. Since no significant differences between the overall effects of conservative caloric restriction therapy and bariatric surgery were detected, decreasing chemerin levels might be causally related to the reduction in body weight and fat mass per se. The data do not indicate a predictive role of chemerin for the achieved extent of body fat loss nor a direct association with type 2 diabetes and liver disease. Among bariatric patients, post-surgical chemerin levels were associated with a reduced risk of liver fibrosis. Future research elaborating on the present data should further elucidate the mechanistic involvement of chemerin in obesity-associated hepatic malfunction and fibrosis. In particular, promising comparative clinical approaches might involve large subsets of severely obese individuals with and without MASLD. Furthermore, characterization of the mechanisms underlying the observed weight loss-associated effects on circulating chemerin remains an issue to be addressed by future investigation.

## Data Availability

The data presented in this study are available on request from the corresponding author.
